# Hyperamylasemia may indicate the presence of ovarian carcinoma: A case report

**DOI:** 10.1097/MD.0000000000013520

**Published:** 2018-12-10

**Authors:** Song Guo, Hongtao Lv, Li Yan, Fengnian Rong

**Affiliations:** Department of Gynecology and Obstetrics, Shandong Provincial Qianfoshan Hospital, Shandong University, Jinan, Shandong, China.

**Keywords:** hyperamylasemia, ovarian carcinoma, surgery

## Abstract

**Rationale:**

Reports of malignant ovarian tumor with hyperamylasemia are very rare. We present a patient with hyperamylasemia who was diagnosed with a malignant ovarian tumor.

**Patient concerns:**

A 46-year-old woman was hospitalized complaining of a 2-day history of abdominal discomfort and fever. On physical examination, she showed abdominal distention and tenderness, with rebound pain. Laboratory evaluation showed significantly elevated serum amylase levels. Computed tomography (CT) revealed a solid mass with uneven density in the pelvis.

**Diagnoses:**

Based on her clinical symptoms and hyperamylasemia, she was suspected to have acute pancreatitis at first. However, the final pathology showed advanced serous papillary ovarian carcinoma.

**Interventions:**

She underwent initial therapy for acute pancreatitis, followed by laparotomy once her symptoms had disappeared. A tumor mass with maximum diameter 12 cm was detected originating from the right ovary, and the patient underwent hysterectomy, bilateral salpingo-oophorectomy with omentectomy, and appendectomy. On the 14th day after the surgery, she received 5 courses of chemotherapy with paclitaxel and carboplatin. However, distant metastasis before the 6th course of chemotherapy were detected by CT, she was therefore changed to a chemotherapy regimen containing gemcitabine and capecitabine.

**Outcomes:**

The final pathology showed advanced serous papillary ovarian carcinoma. On the 14th day after the surgery, she received 5 courses of chemotherapy with paclitaxel and carboplatin. However, her serum CA125 levels rose again before the 6th course of chemotherapy, and CT of the abdomen and pelvis revealed multiple abnormal-density lesions in the peritoneum and pelvic cavity. We considered these to be metastases, and the patient was deemed unresponsive to her previous chemotherapy. She was therefore changed to a chemotherapy regimen containing gemcitabine and capecitabine, and remained on this regimen at the time of writing.

**Lessons:**

Ovarian carcinoma should be considered as a possibility in patients with hyperamylasemia after ruling out other potential common causes. The final diagnosis depends mainly on the clinical manifestation, laboratory results, and CT examination, though pathology is mandatory to confirm the diagnosis. The main treatment is surgical excision.

## 1 Introduction

Ovarian cancer is the leading cause of gynecologic cancer-related deaths among women in the United States.^[1]^ However, the symptoms of ovarian cancer in the early stage are non-specific, and the disease is therefore usually advanced by the time a definite diagnosis is made.^[2]^ Methods that allow the early diagnosis and early treatment of the disease are therefore required. Oncofetal proteins, tumor-associated antigens, such as CA125 and CA199, and enzymes, have been used as tumor markers for the diagnosis of ovarian cancer; however, amylase is an uncommon product of ovarian cancer and amylase-producing ovarian carcinomas are therefore rarely considered or reported. Here we report a rare case of a patient with an amylase-producing ovarian carcinoma.

## 2 Case presentation

**Figure 1:**
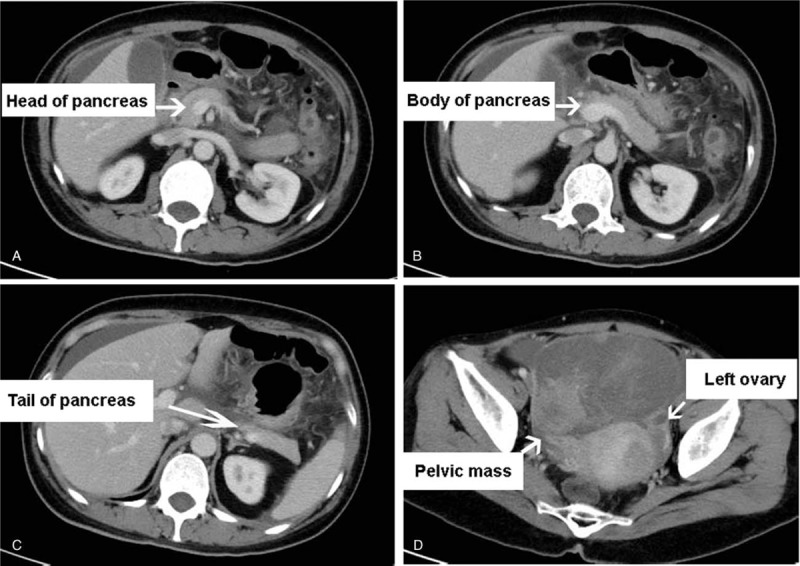
Computed tomography (CT) image of the abdomen and pelvis: a 12 cm-sized solid mass with uneven density in the pelvis (**D**). Normal image of the pancreas (**A**–**C**). CT = computed tomography.

**Figure 2:**
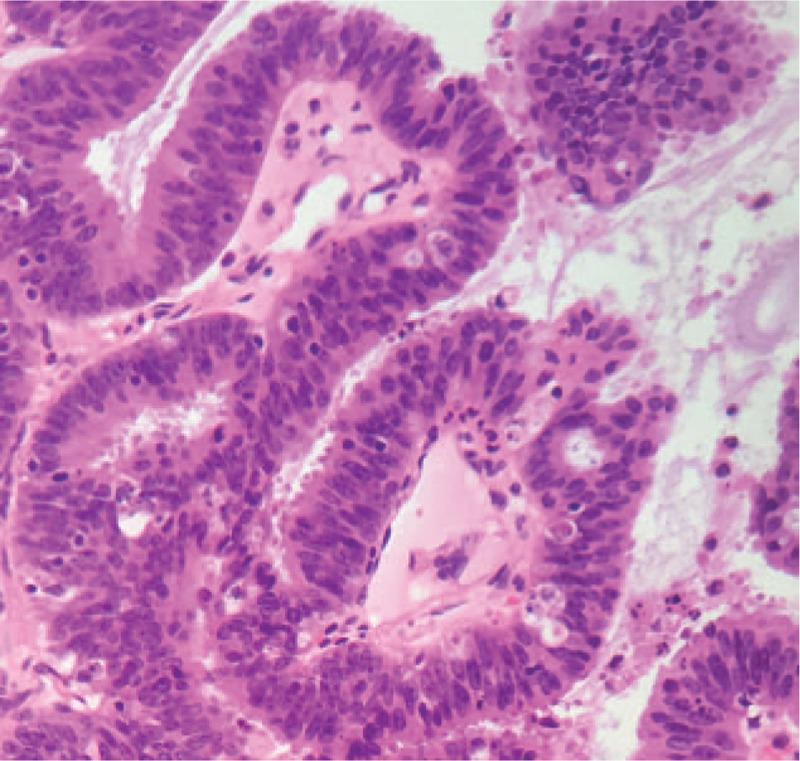
Histology of the right ovary showing serous adenocarcinoma.(×400).

A 46-year-old woman was hospitalized in July 2017 complaining of abdominal discomfort and fever of 2-days duration. She had no family history of malignancy. On physical examination, the patient showed abdominal distention and tenderness, and rebound pain. Abdominal examination also revealed a hard mass in the pelvic cavity, about 10 cm in diameter, located to the upper right of the uterus. Other physical findings were within normal limits. Laboratory evaluation showed raised levels of serum amylase (6713 U/L, normal <110 U/L), CA125 (>1000 U/mL, normal <35 U/mL), and CA19–9 (>1200 U/mL, normal <37 U/mL). Computed tomography (CT) of the abdomen and pelvis revealed a solid mass with uneven density in the pelvis (Fig. 1D). However, the CT scan showed a normal image of the pancreas (Fig. 1**A**–**C**). Based on the overall findings, especially her abdominal pain and raised amylase, acute pancreatitis was suspected and the patient received appropriate therapy, including fasting, decompression, anti-infective treatment, inhibition of pancreatic secretion, and electrolyte balance. Once her symptoms had disappeared, she underwent surgery to obtain a definite diagnosis and to resect the tumor. Laparotomy revealed a tumor mass originating from the right ovary, with a maximum diameter of 12 cm, and a normal pancreas. The patient underwent hysterectomy, bilateral salpingo-oophorectomy with omentectomy, and appendectomy. The pathology results showed advanced serous papillary ovarian carcinoma (Fig. 2). Immunohistochemical examination of the carcinomatous component showed positive immunostaining for p16, Pax-8, p53, progesterone and estrogen receptors, and Ki-67 (60%). The Figo staging of this case is IB.

Four days later, her blood serum amylase dropped back to the normal. Two weeks later, she received 5 courses of chemotherapy with paclitaxel and carboplatin. Her chemotherapy was completed in December 2017 and her CA125 level returned to within the normal range. However, her serum CA125 levels rose again (172.2 U/mL) in January 2018, before the 6th course of chemotherapy, and CT of the abdomen and pelvis revealed multiple abnormal-density lesions in the peritoneum and pelvic cavity. We considered these to be metastases, and the patient was deemed unresponsive to her previous chemotherapy. She was therefore changed to a chemotherapy regimen containing gemcitabine and capecitabine, and remained on this regimen at the time of writing. The patient has provided informed consent for publication of the case.

## 3 Discussion

Raised serum amylase levels occur in many kinds of diseases, and are most commonly associated with pancreatitis, especially in association with acute abdominal pain. Other causes of hyperamylasemia with acute abdominal pain include perforated peptic ulcer, intestinal obstruction or infarction, and diabetic ketoacidosis,^[3]^ but reports of hyperamylasemia in ovarian malignancy are still rare. To the best of our knowledge, the 1st report of an amylase-producing ovarian carcinoma was made by Ende.^[4]^ Amylase is not normally produced by the ovary and ovarian cancer is thus rarely considered as a diagnosis in patients with hyperamylasemia. Here, we report a rare case of a patient with malignant ovarian tumor with hyperamylasemia, highlighting the need to consider ovarian carcinoma as a possible diagnosis in female patients with hyperamylasemia. Monitoring amylase levels may thus also help to make an early diagnosis of ovarian cancer at a more curable stage. In terms of treatment, surgery is the preferred option. Perioperative probing of the pancreas can rule out pancreatitis and pancreas metastasis. The aggressive nature of ovarian tumors means that systemic chemotherapy is usually recommended, even in patients with optimally resected tumors.

The mechanisms responsible for high amylase levels in ovarian carcinoma are still unclear. The current patient showed no pancreatic abnormalities, ruling out pancreatic metastasis, and suggesting that the amylase may have been derived from an extrapancreatic organ. Amylase is encoded by 2 different loci; *AMY1* (salivary-type) and *AMY2* (pancreatic-type),^[5]^ of which the *AMY1* gene mainly encodes non-pancreatic sources of amylase. Sayama et al^[6]^ reported that *AMY1* was exclusively and highly expressed in the salivary glands and in amylase-producing lung adenocarcinomas. Neoplastic transformation of cells may thus result in *AMY1* expression and subsequent biosynthesis of salivary-type amylase. In the current case, the ovarian serous carcinoma may have secreted amylase, similar to lung adenocarcinomas.

Amylase has also been reported to be synthesized directly by the Fallopian tubes, and serous ovarian tumors have been documented to produce amylase from cells that resemble the lining of the Fallopian tube.^[3]^ This may thus provide another biosynthetic pathway for amylase.

Paraneoplastic syndromes are rare disorders caused indirectly by the presence of a malignancy. The carcinoma may cause an inflammatory response resulting in activation and release of the enzyme into the circulation, leading to a series of pathological changes and clinical symptoms. Any organ can be affected, and the clinical symptoms and manifestations thus differ accordingly. Almost every type of cancer, including ovarian cancer, can be associated with paraneoplastic syndromes,^[7]^ and a clinical and pathological study by Hudson et al^[8]^ found that paraneoplastic syndromes were often associated with ovarian cancers. It is therefore reasonable to suppose that amylase production may be associated with ovarian cancer.

The main limitation of this study was that pancreatic amylase expression in the tumor was not confirmed by immunohistochemical staining, making it difficult to evaluate the source of the amylase accurately. Furthermore, this was a single case, and it is therefore difficult to draw any overall conclusions about the features of this disease.

To the best of our knowledge, there have been few previous reports of amylase-producing ovarian carcinomas, and the current case thus provides important information on the clinical behavior of this tumor, and highlights the need for its timely and appropriate treatment. Failure to consider the possibility of this malignancy may lead to delayed treatment, and gynecologists should thus be alerted to the possibility of ovarian carcinoma in patients presenting with hyperamylasemia after ruling out other potential common causes.

## Acknowledgments

This work was supported by the Key Technology Research and Development Programme of Shandong Province (2017G006022).

We thank Susan Furness, PhD, from Liwen Bianji, Edanz Group China (www.liwenbianji.cn/ac), for editing the English text of a draft of this manuscript.

## Author contributions

**Investigation:** Hongtao Lv.

**Writing – original draft:** Song Guo.

**Writing – review & editing:** Li Yan, Fengnian Rong.
